# A Distributed and Energy-Efficient Algorithm for Event K-Coverage in Underwater Sensor Networks

**DOI:** 10.3390/s17010186

**Published:** 2017-01-19

**Authors:** Peng Jiang, Yiming Xu, Jun Liu

**Affiliations:** College of Automation, Hangzhou Dianzi University, Hangzhou 310018, China; xymhdu@163.com (Y.X.); liujunhdu@163.com (J.L.)

**Keywords:** event K-coverage, distributed algorithm, energy-efficient, sensing radius adjusting

## Abstract

For event dynamic K-coverage algorithms, each management node selects its assistant node by using a greedy algorithm without considering the residual energy and situations in which a node is selected by several events. This approach affects network energy consumption and balance. Therefore, this study proposes a distributed and energy-efficient event K-coverage algorithm (DEEKA). After the network achieves 1-coverage, the nodes that detect the same event compete for the event management node with the number of candidate nodes and the average residual energy, as well as the distance to the event. Second, each management node estimates the probability of its neighbor nodes’ being selected by the event it manages with the distance level, the residual energy level, and the number of dynamic coverage event of these nodes. Third, each management node establishes an optimization model that uses expectation energy consumption and the residual energy variance of its neighbor nodes and detects the performance of the events it manages as targets. Finally, each management node uses a constrained non-dominated sorting genetic algorithm (NSGA-II) to obtain the Pareto set of the model and the best strategy via technique for order preference by similarity to an ideal solution (TOPSIS). The algorithm first considers the effect of harsh underwater environments on information collection and transmission. It also considers the residual energy of a node and a situation in which the node is selected by several other events. Simulation results show that, unlike the on-demand variable sensing K-coverage algorithm, DEEKA balances and reduces network energy consumption, thereby prolonging the network’s best service quality and lifetime.

## 1. Introduction and Related Works

Underwater wireless sensor networks (UWSNs) are network monitoring systems that consist of sensor nodes with self-organized perception, acoustic communication, and computation capabilities in an underwater environment. UWSNs can be applied to event detection in underwater environments, such as water pollution monitoring, underwater disaster warnings, and the detection of different types of underwater biology [1,2,3]. Such applications require UWSNs for event detection, as they have strong fault tolerance and robustness. Moreover, UWSNs have a network structure with a 3D distribution, acoustic signals as a communication medium, limited node energy, and a high cost for deployment [4,5]. Because of the limited node energy, energy-efficient performance must be considered when designing algorithms or protocols for wireless sensor networks [6,7,8]. For example, the authors of [9] proposed that the sensor networks should be clustered unevenly to implement energy consumption evenly among cluster head nodes, achieving effective use of the energy of the node; the authors of [10] organized sensors in cooperative groups to reduce the number of messages transmitted inside the network, thereby reducing the energy consumption of the network. Similarly, K-coverage in UWSNs should also consider energy-efficient performance.

K-coverage, in which each target monitoring area (or event) is required to at least be covered by K-sensor nodes (K ≥ 1), can achieve network redundancy detection for events. It is one of the technologies that are commonly used to improve network fault tolerance and robustness [11].

K-coverage has been studied extensively by some researchers [12,13,14,15,16]. Kumar et al. [17] studied how the fewest nodes can be used to ensure network K-coverage at high probability for a monitoring area, for deterministic and stochastic network deployment. Chen et al. [18] proposed a probability-based K-coverage control approach for 3D WSNs. This approach first models the 3D monitoring area by grid. Then, it uses a greedy iteration method to determine the position of each node in the grid until either the node number reaches the preset value or all grid points are covered by K-nodes at a certain probability. Compared with K-coverage methods with deterministic or stochastic network deployment, the algorithm with fewer nodes achieves the same coverage level of a monitoring area, but the overall network coverage could still be improved. Kim et al. [19] proposed a randomly ordered activation and layering protocol for ensuring network K-coverage. This protocol divides all network nodes into several disjoint sets (each disjoint set covers the monitoring area by 1-coverage), i.e., by layering and then randomly selecting K-layer nodes to work together for the network to achieve K-coverage within a certain period. This algorithm can achieve high-coverage level for a monitoring area. Habib M et al. [20] proposed a K-coverage algorithm for a 3D area based on geometric properties. The algorithm firstly analyzes the condition of the 3D area guaranteed to be K-coverage based on Helly’s Theorem and the Reuleaux tetrahedron model, and, on this basis, the corresponding nodes are selected to be active to complete the K-coverage of the network. The algorithm can fully complete K-coverage in a 3D field and increase the lifetime of the network by scheduling the working mode of nodes. A similar idea is studied by Gupta et al. [21]. To prolong the network lifetime, they present a node scheduling strategy for K-coverage. It uses probability to schedule the node to minimize the number of active nodes, according to the number of neighbor nodes and the stage of the node (communication and sensing radius). Pal et al. [22] proposed a K-coverage algorithm based on the Sixsoid. The algorithm finds that using the Sixsoid to fill the 3D area has less overlapping volume than using the Reuleaux tetrahedron model and, according to the characteristic of the Sixsoid, deploys the node in the target area. The algorithm can reduce the number of active nodes, but it can run well also based on the assumption of high density deployment. These algorithms described here can achieve K-coverage for the monitoring area and not the event and will cause numerous idle nodes, thereby wasting energy when they are used in event monitoring. In addition, considering the UWSNs characteristic of high cost and sparse deployment [23], these algorithms, where large numbers of sensor nodes are needed, are unsuitable for event K-coverage for UWSNs.

Several works exist in the literature that covers the target or event on demand by the mobile nodes. Liu et al. [24] uses game theory to determine the optimal mobility strategy of each node to complete the dynamic coverage of the network. Chen [25] presents the energy-effective movement algorithm. The algorithm divides the target area into subareas and then selects nodes to one of the subareas to minimize the movement of nodes. Habib [26] proposed a mission-oriented K-coverage algorithm for mobile WSNs. This algorithm first calculates where the nodes move by using Helly’s theorem and the geometric analysis of the Reuleaux triangle. Then, it adopts the distributed strategy to select a certain number of nodes to move to those positions to achieve K-coverage for the area. The approach reduces the number of nodes deployed in UWSNs and guarantees good event coverage. Xia et al. [27] proposed the fish swarm-inspired underwater sensor deployment algorithm (FSSDA), which calculates the position that nodes will move to by the method inspired by the fish feeding behavior, and combines the position with the crowded degree control to achieve coverage requirements for the events. The algorithm has low complexity and a quick convergence rate, but it considers only the required coverage for static events and ignores the dynamic and uncertainty of the events. Du et al. [28] proposed the particle swarm-inspired underwater sensor deployment algorithm, which is similar to the FSSDA, and considers event dynamic to make the algorithm more practical. Although the algorithm is the same as other event K-coverage algorithms by mobile nodes, it greatly reduces the number of nodes in network deployment. However, it also consumes considerable energy because of the nodes’ mobility [29]. Alam et al. [30] proposed the on-demand variable sensing K-coverage algorithm (OVSKA), which implements event dynamic K-coverage in the network by adjusting the sensing radius of node and analyzes its own practical application. The OVSKA better reduces the number of nodes and redundant energy consumption by the dynamic event K-coverage method compared with the static method. It also effectively avoids energy consumption produced by node mobility by adjusting the sensing radius. However, for how to select the assistant nodes to achieve the K-coverage for each event, the paper only proposed a greedy algorithm, in which each management node selects its assistant nodes from its neighbor nodes to minimize the ratio of network energy consumption of its neighbor nodes to detection performance of the events it manages. The process of selecting adjusting nodes is simple. However, when the number of events is relatively large, the neighbor node of the management node can cover some events, which several management nodes manage by adjusting the sensing radius. At this time, in the method, a management node cannot consider a situation in which other management nodes may select its neighbor node to cover the event they manage. As a result, the number of nodes that broaden their sensing radius increases and network energy consumption to some extent increases. In addition, the method selects the assistant node, ignoring the residual energy of the node and causing some nodes to be scheduled frequently and die early. This approach makes the network energy consumption unbalanced, degrades the quality of network service, and shortens network lifetime.

To solve the aforementioned problems, this study proposes the distributed and energy-efficient event K-coverage algorithm (DEEKA). The monitoring area achieves 1-coverage or approximately 1-coverage. At the start of each round of the algorithm, nodes that detect the same event compete for the management node of the event by the indicator, including the number of candidate nodes, the average residual energy of neighbor nodes, and the distance to the event. Each management node then calculates the probability of each neighbor node’s being selected by the corresponding event it manages. Then, each management node builds a multi-objective optimization model with regard to the energy consumption expectation and residual energy variance of its neighbor nodes, and to the detected performance for the events it manages as targets. Afterwards, each management node obtains Pareto solutions by using the constrained elitist non-dominated sorting genetic algorithm (NSGA-II) method and selects the best strategy by using the technique for order preference by similarity to an ideal solution (TOPSIS) method according to the target bias of practical application. The algorithm can make some nodes adjust the sensing radius to cover events that different management nodes manage synchronously, thereby reducing the number of nodes that increase their sensing radius and energy consumption over the whole network. In addition, determining management nodes by competition is beneficial for balancing residual energy of the neighbor nodes among different management nodes. When each management node selects its assistant nodes, it considers the residual energy of its neighbor nodes. This approach is beneficial for balancing energy consumption of its neighbor nodes. Simulation results show that, compared with the OVSKA, the DEEKA can better balance and reduce network energy consumption, thereby prolonging the network’s best service quality and lifetime.

The rest of this paper is organized as follows: Section 2 describes the system model, assumptions, and definitions considered in this study. Section 3 presents details of the DEEKA. Section 4 analyzes the complexity of the DEEKA. Section 5 discusses the performance study and provides a detailed analysis of its result. Finally, Section 6 concludes the paper and presents future research directions.

## 2. Preliminaries: Models and Definitions

### 2.1. Models

#### 2.1.1. Network Model

**Figure 1:**
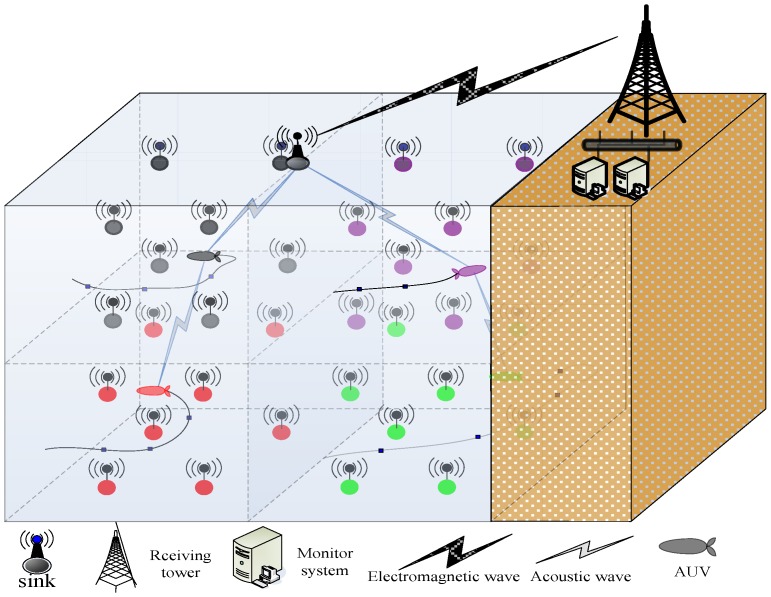
Underwater wireless sensor network (UWSN) system model.

A monitoring area achieves 1-coverage or approximately 1-coverage by *N* nodes. Meanwhile, these nodes are anchored with weights to keep static in current positions. It is evenly divided into several sub-areas. Additionally, it has a mobile intermediate node (e.g., Autonomous Underwater Vehicle, AUV) that serves as the router that receives information from management nodes and then transmits that information to the sink node in every sub-area [31]. Every assistant node adjusts to the appropriate sensing radius to help the corresponding management node monitor the event together and sends the event sensing information to the management node. The management node obtains the result about the event by using the fusion algorithm and waits for the mobile intermediate node to gather this information. After the event leaves or disappears, the corresponding assistant nodes revert to the minimum sensing radius. The network model is depicted in Figure 1. Every AUV is responsible for handling the information of the management node with the same color as the AUV and for transmitting the information to the sink node.

This study denotes the *i*-th node by *s_i_*, and the corresponding node set $S=\left\{s_{1},s_{2},\ldots s_{n}\right\}$, as well as the *j*-th event by *e_j_* and the corresponding event set $E=\left\{e_{1},e_{2},\ldots e_{z}\right\}$. Moreover, the number of elements in the set *S* is *N* and that in the set *E* is *Z*. The following assumptions are considered:

1. Any node has the ability of communication and perception. Nodes communicate with one another through acoustic channels, and the sink node communicates with the ground monitoring station by radio.
2. All nodes are isomorphic before the algorithm runs. Then, the sensing radius of each node can be adjusted between the minimum $R_{s}^{\min}$ and maximum sensing radius $R_{s}^{\max}$ according to adjustment strategy.
3. The position of each event is randomly changed by the water current in the monitoring area but not beyond that. Time interval *T* is one round of network run. In every round, the algorithm readjusts the sensing radius of some corresponding nodes to achieve K-coverage for all events whose position is changed.

#### 2.1.2. Network Energy Consumption Model

In sensor networks, each node is mainly responsible for sensing the surrounding environment and transmitting the sensing data. Therefore, in this study, the node’s energy consumption includes sensing and communication [32,33].

##### Energy Consumption Model of Communication

Underwater sensor network nodes communicate with one another through acoustic signals. Accordingly, this study uses the energy consumption model of underwater sensor network data communication by utilizing sound wave as the medium [33]. Underwater acoustic signal attenuation model *SA*(*d*) is given as follows:

(1) $$SA(d)=d^{\lambda }\cdot \alpha^{d}.$$

Equation (1) describes energy attenuation when the transmission distance of data packet is *d*, where *λ* is the energy diffusion factor (cylindrical diffusion is 1, actual situation is 1.5, and spherical diffusion is 2). Parameter *α =* 10*^AC^*^(*f*)*/*10^, which is determined by absorption coefficient *AC*(*f*), which is shown as follows:

(2) $$AC(f)=0.11\frac{10^{-3}f^{2}}{1+f^{2}}+44\frac{10^{-3}f^{2}}{4100+f^{2}}+2.75\times 10^{-7}f^{2}+3\times 10^{-6}$$

where *f* is the carrier frequency with the kHz unit. Absorption coefficient unit is dB/m.

The energy consumption of communication on the distance *d* (*ECC*(*d*)) is expressed as follows:

(3) $$ECC(d)=P_{r}\times T_{p}\times SA(d)$$

where *T_p_* is the data transmission time, and *P_r_* is the minimum power packets that can be received.

##### Energy Consumption Model of Sensing

According to Reference [34], the ratio of sensing power to communication power is *r_p_* (0 < *r_p_* < 1). Thus, in this study, the energy consumption model of sensing on the distance *d* *ECS*(*d*) is shown as follows:

(4) $$ECS\left(d\right)=r_{p}\times P_{r}\times SA\left(d\right)\times T.$$

*T* is the time interval of the algorithms running.

#### 2.1.3. Node Sensing Model

At present, the sensing model for the event can be divided into two categories: the Boolean sensing model and the probability sensing model. The former is unsuitable for practical applications because it is too idealistic. Therefore, this study uses the improved probability sensing model, which considers sensing ability characteristics of the sensor and interference characteristics of noise in the environment.

At first, the model considers sensor characteristics, i.e., the sensing performance of detecting the abnormal signal, including event and noise signals, which is good within a certain range but degrades with distance beyond the range [35]. Sensing probability of an event is formulated as follows:

(5) $$p\left(d\left(s_{i},e_{j}\right)\right)=\left\{ \begin{array}{l} 1\;\mathrm{if}\;d\left(s_{i},e_{j}\right)\leq R_{s}^{\min}\\ e^{-\gamma_{1}{\left(d\left(s_{i},e_{j}\right)-R_{s}^{\min}\right)}^{\gamma_{2}}}\;\mathrm{if}\;R_{s}^{\min}<d\left(s_{i},e_{j}\right)<R_{s}^{\max}\\ 0\;\mathrm{if}\;d\left(s_{i},e_{j}\right)\geq R_{s}^{\max}. \end{array}\right.$$

where $\gamma_{1}$ and $\gamma_{2}$ are specific parameters of the sensing device that are determined from physical properties of the sensor. They determine the speed of sensing probability decay beyond $R_{s}^{\min}$.

The judgment result of one sensor node to the event is vulnerable to environmental noise (i.e., noise signal). Thus, the influence of environmental noise on the current sensing probability of an event should be considered. Assuming that the noise signal can be expressed by Gaussian distribution *Ψ*(0,1), whose average value is 0 and variance is 1, the abnormal signal threshold for event detection is *η_t_*. When the signal strength is lower than *η_t_*, the probability that the event is correctly sensed by the node reduces quickly. Therefore, the probability *P*(*s_i_*,*e_j_*) that an event *e_j_* is correctly sensed by node *s_i_* is formulated as follows [30]:

(6) $$P\left(s_{i},e_{j}\right)=p\left(d\left(s_{i},e_{j}\right)\right)\int_{\eta_{t}}^{\infty }\frac{1}{\sqrt{2\pi }}e^{-{\left(x-u\left(s_{i}\right)\right)}^{2}/2}\;dx$$

where *u*(*s_i_*) is the abnormal signal strength (including event and noise signals) node *s_i_* receives, and *d*(*s_i_*,*e_j_*) is the distance between *s_i_* and *e_j_*.

#### 2.1.4. Event Mobility Model

The event randomly moves after it appears within the monitoring area through the water current. Assuming that the position coordinate of the event at *t* time is (*x*(*t*), *y*(*t*), *z*(*t*)), the event mobility model is

(7) $$\begin{array}{l} x(t)=x(t-1)+d_{x}\left(t\right)v_{cx}\\ y(t)=y(t-1)+d_{y}\left(t\right)v_{cy}\\ z(t)=z(t-1)+d_{z}\left(t\right)v_{cz} \end{array}$$

where

$$d_{x}\left(t\right)=\left\{ \begin{array}{l} 1\;t=0\\ -d_{x}\left(t-1\right)\;t>0 \end{array}\right.,\;d_{y}(t)=\left\{ \begin{array}{l} 1\;t=0\\ -d_{y}\left(t-1\right)\;t>0 \end{array}\right.,\;d_{z}(t)=\left\{ \begin{array}{l} 1\;t=0\\ -d_{z}\left(t-1\right)\;t>0 \end{array}\right..$$

*v_cx_*, *v_cy_*, and *v_cz_* are the flow speed of *x*-axial direction, *y*-axial direction, and *z*-axial direction, respectively. Their values are all randomly produced between 0 and maximum speed *V*_max_.

### 2.2. Definition

#### 2.2.1. Nodes and Events

**Neighbor Node:** A node, whose distance to the node *s_i_* is within its communication radius *R_c_*, is *s_i_*’s neighbor node, and all of *s_i_*’s neighbor nodes form the set of the neighbor node, denoted by *S_N_*(*s_i_*).

**Figure 2:**
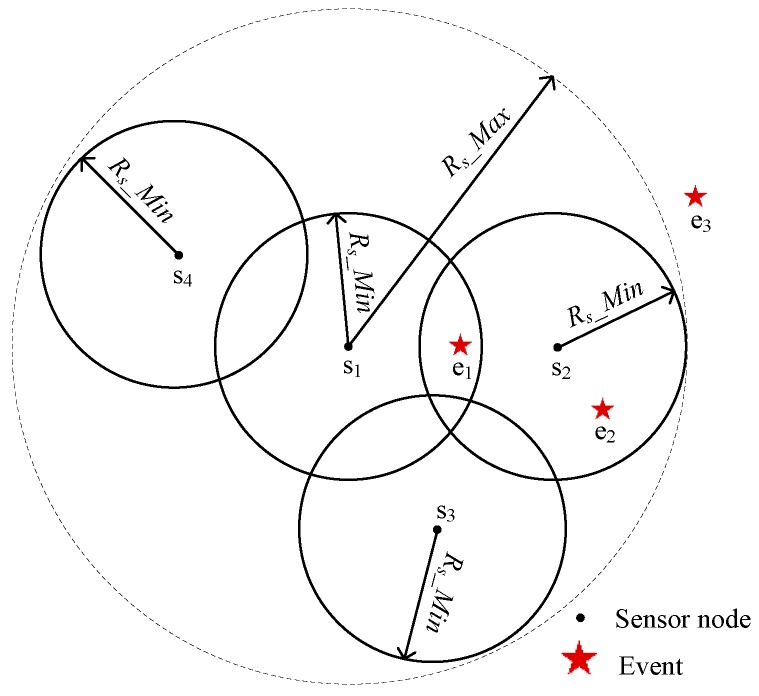
Sectional view of the relationship between nodes and events.

**Management Node:** The management node of one event *e_j_* is produced as discussed in Section 3.2.1 and is responsible for scheduling its corresponding neighbor nodes to cover *e_j_* on demand. In other words, *e_j_* is an event that the management node manages. In addition, all of the management nodes form the set of the management node, denoted by *M*, where *M* = {*m*_1_, *m*_2_, … *m_m_*}, and *m_m_* is the *m*-th element of *M*, namely, the *m*-th management node. Similarly, all of the events that *m_m_* manages form the set denoted by *E_M_*(*m_m_*). In Figure 2, *s*_2_ is the management node of *e*_2_; the management node of *e*_1_ is generated from the competition between *s*_1_ and *s*_2_.

**Candidate Node:** The candidate node of one event *e_j_* is defined as the node in the neighbor node set of *e_j_*’s management node *S_N_*(*m_m_*), which can adjust its sensing radius to cover *e_j_*. In other word, the candidate node of *e_j_* is in the neighbor nodes of *e_j_*’s management node *m_m_*, and its distance to *e_j_* is less than $R_{s}^{\max}$. Therefore, when the management node of *e_j_* is *m_m_*, the set of the candidate node of *e_j_* can be denoted by *m_m_*.*C*(*e_j_*) and formulated as follows:

(8) $$m_{m}.C\left(e_{j}\right)=\left\{s_{i}|d\left(s_{i},e_{j}\right)<R_{s}^{\max}\;and\;s_{i}\in S_{N}\left(m_{m}\right)\right\}.$$

Because the management node of each event is determined in the study, except in Section 3.2.1, the study uses *C*(*e_j_*) to replace *m_m_*.*C*(*e_j_*) for simplified representation. Needless to say, the number of *e_j_*’s candidate nodes is the number of elements in *C*(*e_j_*), denoted by |*C*(*e_j_*)|. In Figure 2, candidate nodes of *e*_1_ and *e*_2_ for events *e*_1_ and *e*_2_ are both *s*_1_ and *s*_2_.

**Dynamic Candidate Node:** According to the description of the candidate node, the node whose distance to an event *e_j_* is between $R_{s}^{\min}$ and $R_{s}^{\max}$ is defined as the dynamic candidate node, and the kind of the node forms the set of the dynamic candidate node, denoted by *C_d_*(*e_j_*):

(9) $$C_{d}\left(e_{j}\right)=\left\{s_{i}|R_{s}^{\min}<d\left(s_{i},e_{j}\right)\leq R_{s}^{\max},\;and\;s_{i}\in C\left(e_{j}\right)\right\}$$

where the *i*-th element in *C_d_*(*e_j_*) is assigned as $c_{d}^{i}$. Clearly, the number of dynamic candidate nodes of *e_j_* is denoted by |*C_d_*(*e_j_*)|. As shown in Figure 2, the dynamic candidate node for event *e*_1_ is *s*_3_, and, for event *e*_2_, dynamic candidate nodes are *s*_1_ and *s*_3_.

**Static Candidate Node:** Similarly, the static candidate node of an event *e_j_* is the node in *e_j_*’s candidate node set, whose distance to *e_j_* is within $R_{s}^{\min}$. Correspondingly, the kind of the node forms the set of the static candidate node, denoted by *C_s_*(*e_j_*) and formulated as follows:

(10) $$C_{s}\left(e_{j}\right)=\left\{s_{i}|d\left(s_{i},e_{j}\right)\leq R_{s}^{\min},\;and\;s_{i}\in C\left(e_{j}\right)\right\}$$

where the *i*-th element in *C_s_*(*e_j_*) is assigned as $c_{s}^{i}$. Clearly, the number of dynamic candidate nodes of *e_j_* is denoted by |*C_s_*(*e_j_*)|. In Figure 2, the static candidate node for event *e*_1_ is *s*_1_, while event *e*_2_ does not have a static candidate node.

The difference between the dynamic candidate node and the static candidate node is the distance between the node and the event. Because of the difference, the former needs to adjust its sensing radius to cover the event. However, the latter does not need to do this.

**Assistant Node:** The assistant node of one event *e_j_* is defined as the node, which is scheduled by the management node and assists the management node to cover *e_j_*. In this paper, the assistant node is also described as selected by *e_j_*, and all of the nodes form the set of the assistant node, denoted by *A*(*e_j_*).

Whether or not the sensing radius is adjusted, the assistant node can be divided into two categories: dynamic and static. The dynamic assistant node assists the management node to detect the event by increasing its sensing radius. Conversely, the static assistant node does not need to increase its sensing radius. In Figure 2, *s*_1_ and *s*_3_ are the selected assistant nodes of event *e*_1_. Between them, *s*_1_ is the static assistant node of *e*_1_, and *s*_3_ is its dynamic assistant node.

**Static Coverage Event:** The static coverage event of one node *s_i_* is defined as an event whose distance to *s_i_* is within minimum sensing radius $R_{s}^{\min}$, and all of the events form the set of the static coverage event, denoted by *E_s_*(*s_i_*) and formulated as follows:

(11) $$E_{s}\left(s_{i}\right)=\left\{e_{j}|d\left(s_{i},e_{j}\right)\leq R_{s}^{\min}\right\}.$$

Clearly, the number of *s_i_*’s static coverage event is the number of elements in *E_s_*(*s_i_*), denoted by |*E_s_*(*s_i_*)|. In Figure 2, the static coverage event for node *s*_1_ is *e*_1_.

**Dynamic Coverage Event:** The static coverage event of one node *s_i_* is defined as an event whose distance to *s_i_* is between $R_{s}^{\min}$ and $R_{s}^{\max}$, and all of the events form the set of the dynamic coverage event, denoted by *E_d_*(*s_i_*) and formulated as follows:

(12) $$E_{d}\left(s_{i}\right)=\left\{e_{j}|R_{s}^{\min}<d\left(s_{i},e_{j}\right)\leq R_{s}^{\max}\right\}.$$

Clearly, the number of *s_i_*’s dynamic coverage event is the number of elements in *E_d_*(*s_i_*), denoted by |*E_d_*(*s_i_*)|. In Figure 2, *e*_1_ is the static coverage event of node *s*_1_. Both *e*_1_ and *e*_2_ are the dynamic coverage events of *s*_3_.

The difference between the static coverage event and the dynamic coverage event can be summarized as follows. As shown in Equation (11), the event can be sensed by the node directly, because the distance between them is within the minimum sensing radius. Thus, the kind of the node is named after the static coverage event. However, the event described in Equation (12) wants to be covered by the node, which needs the node to adjust its sensing radius. Thus, the kind of the node is named after the dynamic coverage event.

**Actual Dynamic Coverage Event:** The actual dynamic coverage event is the event that *s_i_* actually adjusts its sensing radius to cover after the algorithm runs, and the set is assigned as *E_ad_*(*s_i_*). The element number of the set is assigned as |*E_ad_*(*s_i_*)|.

The difference between the dynamic coverage event and the actual dynamic coverage event is that an event is the dynamic coverage event of *s_i_*, but it does not have to be the actual dynamic coverage event of *s_i_*. Therefore, the number of actual dynamic coverage events is usually less than that of dynamic coverage events.

#### 2.2.2. Event Detection Performance

Event detection performance reflects the quality of the network service and the robustness of the network to detect events. It is defined as the correct sensing probability of event *e_j_*. Event detection performance is judged by information fusion center (i.e., the management node in this study) under the circumstance that information may be lost in the transmission process because of the influence of some interference in practice. The information fusion center uses the rule of *k*_1_ out of K [11]. Thus, it considers event *e_j_* to be detected correctly when at least *k*_1_ nodes in K-nodes detect *e_j_* correctly. Assuming that the failure probability of information transmission between two nodes is *p_lost_*, the probability that the information of node *s_i_* correctly detecting *e_j_* is successful to be transmitted to the fusion center is expressed as follows and is denoted by *P_reach_*(*s_i_*,*e_j_*):

(13) $$P_{reach}\left(s_{i},e_{j}\right)=\left(1-p_{lost}\right)\cdot P\left(s_{i},e_{j}\right).$$

The probability of the fusion center correctly detecting event *e_j_* is formulated and is denoted by *P_result_*(*e_j_*).

(14) $$P_{result}\left(e_{j}\right)=\sum_{b=k_{1}}^{K}\left[\sum_{C_{K,b}}\left(\prod_{i=1}^{b}P_{reach}\left(a_{i},e_{j}\right)\prod_{i=b+1}^{K}\left(1-P_{reach}\left(a_{i},e_{j}\right)\right)\right)\right]$$

where *C*_K*,b*_ is the value of combinations of *b* nodes in K-nodes correctly detecting events; *a_i_* is *i*-th elements in *e_j_*’s assistant node set *A*(*e_j_*); the order of the element in *A*(*e_j_*) is changed by different combinations. In each combination, the nodes that detect the event correctly are in the front, while others are at the back.

#### 2.2.3. Network Lifetime

Network lifetime is an important basis for evaluating the effectiveness of the energy algorithm [36] and is denoted by *L_t_*. In this study, the network lifetime is defined as the value of the network running round until the ratio of the number of survival nodes to the total number of nodes achieves *r_n_* (0 ev*r_n_* < 1), where *r_n_* is the proportional threshold of surviving nodes. When the ratio is less than *r_n_*, the network cannot complete normal monitoring function, and network running ends.

## 3. Algorithm Description and Process

### 3.1. Problem Description

Some research has been conducted on the K-coverage problem of sensor networks [12]. Most existing algorithms for static K-coverage of an area need numerous nodes. The existing algorithms also cause great redundant energy consumption for event K-coverage in UWSNs. Others achieve event K-coverage by assisting mobile nodes, which consumes considerable energy because of nodes moving. A dynamic event K-coverage method, by adjusting the sensing radius of nodes, can reduce the required number of deployment nodes. This approach also considerably reduces the redundant energy consumption produced by the moving nodes. For assistant node selection, each management node selects the corresponding assistant nodes to achieve K-coverage and minimizes the ratio of network energy consumption to the detecting performance of the event it manages. The method can achieve a relatively good result when the neighbor node of the management node can only cover the event that the management node manages on its own. However, when the number of events is relatively large, the neighbor node can usually cover more than one event that different management nodes manage. At this condition, the method will also increase the number of nodes that adjust their sensing radius in the network and the energy consumption of the network. In addition, each management node in the algorithm ignores the residual energy of its neighbor nodes when it selects the assistant nodes. As a result, the sensing radius of some nodes increases frequently, thereby causing network energy consumption imbalances, degrading the quality of network service, and reducing network lifetime. Therefore, after the network achieves 1-coverage or approximately 1-coverage by *N*-nodes, an energy-efficient event K-coverage algorithm should be designed to achieve dynamic K-coverage for UWSNs, balance and reduce network energy consumption, prolong network lifetime, and increase network K-detecting performance.

This study proposes the DEEKA to solve this problem. The monitoring area achieves 1-coverage or approximately 1-coverage. First, at the start of each round of the algorithm, nodes that detect the same event compete for the management node of the event by the indicator, including the number of candidate nodes, the average residual energy of neighbor nodes, and the distance to the event. This process balances residual energy among neighbors with different management nodes and considers network detecting performance. Second, each management node calculates the probability of its neighbor nodes’ being selected by the corresponding event it manages, according to levels of distance and residual energy of nodes in the set of the dynamic candidate node, as well as the number of dynamic coverage events of the node. This process is beneficial for each management node in that its neighbor nodes may dynamically cover several events when it selects assistant nodes. Moreover, it is beneficial for each management node to select synchronously and achieve distributed implementation of the algorithm. Third, each management node builds a multi-objective optimization model of the energy consumption expectation of its neighbor nodes, the residual energy variance of its neighbor nodes, and the detection performance of the events it manages as targets. Afterwards, the management node obtains Pareto solutions by using a constrained NSGA-II method. This process allows each management node to obtain all the best solutions (namely, node selection strategy) with three objectives constrained with one another. It is also beneficial for algorithm extension. Finally, *m_m_* selects the best strategy using the TOPSIS method according to the target bias of practical application. Corresponding nodes adjust their sensing radius to achieve K-coverage for the event. A detailed description is presented below.

### 3.2. Algorithm Description

The DEEKA is divided into the following four steps: (1) management node formation; (2) calculation probability of an event-selected node; (3) a multi-objective optimization model; (4) a methodology of a constrained NSGA-II and optimization strategy.

#### 3.2.1. Management Node Formation

At the beginning of each round of the algorithm, dynamical assistant nodes at the last round broadcast their residual energy. Each node updates the state information of its neighbor node, including residual energy and survival node number (the first round of the algorithm ignores this step). After the nodes detect the event (e.g., *e_j_*), each node that detects *e_j_* (e.g., *s_i_*) calculates the distance between its neighbor nodes and *e_j_*. Then, it compares the distance with the maximum sensing radius to produce the candidate node set of *e_j_* within *s_i_* (*s_i_*.*C*(*e_j_*) ) and *s_i_*.|*C*(*e_j_*)|. *S_i_* then broadcasts message *I_c_* (node ID, ID of events it detects, and average residual energy of nodes in *s_i_*.*C*(*e_j_*) and *s_i_*.|*C*(*e_j_*)|). After *s_i_* receives *I_c_* from other nodes (e.g., *s_m_*), it saves the related information of other nodes who also detect *e_j_* and then determines if it becomes the management node of *e_j_* according to the cases as follows:

- If *s_i_*.|*C*(*e_j_*)| is less than K − 1 and the candidate node number of the other nodes detecting *e_j_* is also less than K − 1, *s_i_* judges its |*C*(*e_j_*)| among nodes detecting *e_j_*. If *s_i_*.|*C*(*e_j_*)| is not the largest, it automatically gives up the competition of the management node; otherwise, it becomes the management node of *e_j_* and joins the management node set *M,* as well as skips the rest of the process described in this section.
- If *s_i_*.|*C*(*e_j_*)| is less than K − 1 and the candidate node number of the other nodes detecting *e_j_* is not all less than K − 1, *s_i_* gives up the competition of management node.
- If *s_i_*.|*C*(*e_j_*)| is greater than K − 1, *s_i_* then calculates its score *Score*_1_(*s_i_*) and other nodes’ score *Score*_1_(*s_m_*) by Equation (15). It calculates according to the indicators of the average residual energy of and number of candidate nodes, and the distance to *e_j_*. *Score*_1_(*s_i_*) is formulated as follows:
  (15) $$Score_{1}\left(s_{i}\right)=w_{1}\frac{RE_{av}\left(s_{i},e_{j}\right)-RE_{av}^{\min}}{RE_{av}^{\max}-RE_{av}^{\min}}+w_{2}\frac{d_{\max}-d\left(s_{i},e_{j}\right)}{d_{\max}-d_{\min}}+w_{3}\frac{s_{i}.\left|C\left(e_{j}\right)\right|-{\left|C\left(e_{j}\right)\right|}_{\min}}{{\left|C\left(e_{j}\right)\right|}_{\max}-{\left|C\left(e_{j}\right)\right|}_{\min}}$$
  where *RE_av_*(*s_i_*,*e_j_*) is the average residual energy of *s_i_*’s candidate node for *e_j_*, $RE_{av}^{\max}$ and $RE_{av}^{\min}$ are the maximum and minimum average residual energy of the candidate node of nodes detecting *e_j_*, respectively; *d*_max_ and *d*_min_ are the maximum and minimum distances to *e_j_*, respectively; ${\left|C\left(e_{j}\right)\right|}_{\max}$ and ${\left|C\left(e_{j}\right)\right|}_{\min}$ are the maximum and minimum numbers of the *e_j_* candidate node, respectively.
  *s_i_* then compares its score with others. If its score is not the greatest, then *s_i_* gives up the competition for the management node of *e_j_*. Otherwise, *s_i_* becomes the management node of *e_j_* and joins management node set *M*.
- If the information *s_i_* receives does not include event *e_j_*, which is to say, only *s_i_* detects *e_j_*, *s_i_* becomes the management node of *e_j_* and joins management node set *M*.

**Figure 3:**
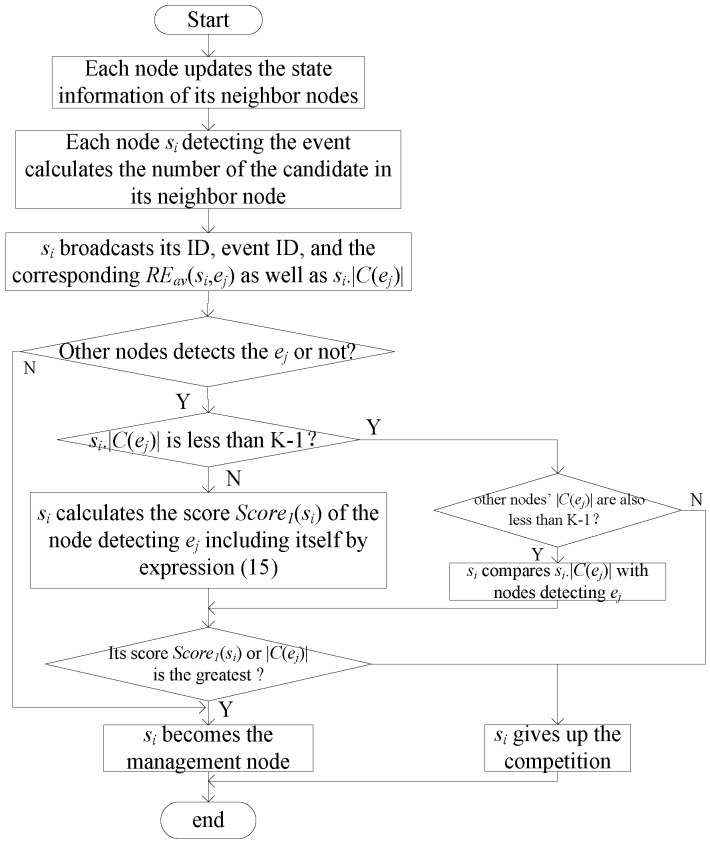
Flowchart of Section 3.2.1.

The flowchart of the management node of each round is shown in Figure 3, with event *e_j_* as an example.

#### 3.2.2. Calculation Probability of Event-Selected Node

After the management node is determined, each management node (e.g., *m_m_*) broadcasts a helping message (node ID) with the communication radius *R_c_*. Each node that receives the helping message sends its state message (node ID and number of dynamic coverage event |*E_d_*(*s_i_*)|) to the corresponding management node. After node *m_m_* receives the state message, it calculates the distance between its neighbor nodes (e.g., *s_i_*) and each event it manages (e.g., *e_j_*). Then, it compares the distance with the minimum sensing radius $R_{s}^{\min}$. If *d*(*s_i_*,*e_j_*) ≤ $R_{s}^{\min}$, *s_i_* joins into static candidate node set *C_s_*(*e_j_*). Otherwise, *s_i_* joins dynamic candidate node set *C_d_*(*e_j_*). If $\left|C_{s}\left(e_{j}\right)\right|$ is greater than K − 1, then the probability of the node in *C_d_*(*e_j_*) being selected by *e_j_* is 0. That is to say, to achieve K-coverage for *e_j_*, none of the nodes need to adjust its sensing radius. Thus, *m_m_* skips *e_j_*, removes it out of *E_M_*(*m_m_*), and calculates the probability of the node selected by other events it manages, namely, for *e_j_*, it skips the rest of the process described in this section. Otherwise, *m_m_* then calculates levels of residual energy *L_RE_*($c_{d}^{i}$) and distance *L_d_*($c_{d}^{i}$) of each dynamic candidate node (e.g., $c_{d}^{i}$) in *C_d_*(*e_j_*). The process is shown as follows:

(16) $$L_{RE}\left(c_{d}^{i}\right)=\frac{RE\left(c_{d}^{i}\right)-RE_{\min}}{RE_{\max}-RE_{\min}},\;L_{d}\left(c_{d}^{i}\right)=\frac{d_{\max}-d\left(c_{d}^{i},e_{j}\right)}{d_{\max}-d_{\min}}$$

where *RE*($c_{d}^{i}$) is the residual energy of dynamic candidate node $c_{d}^{i}$; *RE*_min_ and *RE*_max_ are the minimum and maximum residual energy among nodes in *C_d_*(*e_j_*), respectively; *d*_min_ and *d*_max_ are the minimum and maximum distances to *e_j_* among nodes in *C_d_*(*e_j_*), respectively.

According to the number of its dynamic coverage event $\left|E_{d}\left(c_{d}^{i}\right)\right|$, *L_RE_*($c_{d}^{i}$), and *L_d_*($c_{d}^{i}$), *m_m_* then estimates the probability that $c_{d}^{i}$ is selected to assist it to detect *e_j_*, namely, *e_j_*’*s* assistant node. The number of $c_{d}^{i}$’s actual dynamic coverage event *E_ad_*($c_{d}^{i}$) is usually less than $\left|E_{d}\left(c_{d}^{i}\right)\right|$, and it is not a certain value before the algorithm run. Therefore, *m_m_* considers a situation in which $c_{d}^{i}$ is selected by *e_j_* with different combinations of the actual coverage event number, which is produced by the dynamic coverage event number of nodes in *c_d_*(*e_j_*), and *m_m_* then estimates the probability of the node’s being selected by *e_j_*. In other words, *m_m_* produces all the different combinations, and every combination consists of a number within the dynamic coverage event number of each node in *C_d_*(*e_j_*). For example, if $c_{d}^{1},c_{d}^{2},c_{d}^{3}$ are the dynamic candidate nodes in *C_d_*(*e_j_*), and *|E_d_*($c_{d}^{1}$)*|*, *|E_d_*($c_{d}^{2}$)*|*, and *|E_d_*($c_{d}^{3}$)*|* are 1, 2, and 3, respectively, then all of the combinations of the actual dynamic coverage event are (0,0,0), (0,0,1), (0,0,2), (0,0,3), (0,1,0), (0,1,1), …, etc. Then, *m_m_* determines $c_{d}^{i}$ is selected or not according to Equation (17) for each combination and finally regards the frequency of $c_{d}^{i}$’s being selected as the probability of $c_{d}^{i}$’s being selected by *e_j_*.

The total number of combination increases in factorial form with $\left|E_{d}\left(c_{d}^{i}\right)\right|$ and |*C_d_*(*e_j_*)|. Thus, this study estimates the probability of $c_{d}^{i}$’s being selected by *e_j_* by sampling the part among the total combinations. In order to improve the accuracy of the probability, repeat the above process one round and the average of the result of total rounds is regarded as the probability of $c_{d}^{i}$’s being selected by *e_j_*. According to Reference [37], when the sample size is greater than 10,000,000, the sample number is 400 with a 0.05 margin of error, and the confidence level is 95%. Thus, in this study, the sample number is 400 regardless of the sample size.

(1) For each node $c_{d}^{i}$, *m_m_* randomly selects an integer in the interval [0, *|E_d_*($c_{d}^{i}$)*|* − 1] as the number of $c_{d}^{i}$’s actual dynamic coverage events, and these integers that *m_m_* selects form a combination of the number of actual coverage events, namely, one sampling.
(2) *m_m_* calculates the score *Score*_2_($c_{d}^{i}$) of each node $c_{d}^{i}$ in the current combination by Equation (17), according to *L_RE_*($c_{d}^{i}$), *L_d_*($c_{d}^{i}$), and $\left|E_{ad}\left(c_{d}^{i}\right)\right|$:
  (17) $$Score_{2}\left(c_{d}^{i}\right)=w_{1}L_{RE}\left(c_{d}^{i}\right)+w_{2}L_{d}\left(c_{d}^{i}\right)+w_{3}\frac{\left|E_{ad}\left(c_{d}^{i}\right)\right|-E_{ad}^{\min}}{E_{ad}^{\max}-E_{ad}^{\min}}$$
  where *w*_1_, *w*_2_, and *w*_3_ are indicator weights whose values are set according to the application focusing on the relevant indicators and *w*_1_ + *w*_2_ + *w*_3_ = 1; $E_{ad}^{\min}$ and $E_{ad}^{\max}$ are the minimum and maximum numbers of the actual coverage events in the current combination, respectively.
(3) *m_m_* selects K – 1 − $\left|C_{s}\left(e_{j}\right)\right|$ nodes as the dynamic assistant nodes of *e_j_*, according to the scores in descending order (nodes in $C_{s}\left(e_{j}\right)$ must be the assistant nodes of *e_j_* because they do not need to increase their sensing radius to cover *e_j_*). Other nodes are not selected in the current combination.
(4) If the sample number is less than 400, then Step 1 is repeated. Otherwise, the next step is performed.
(5) For each node $c_{d}^{i}$, *m_m_* records and calculates the frequency of $c_{d}^{i}$’s being selected to cover *e_j_ Frequency*($c_{d}^{i}$, *e_j_*) in the current round of sampling.

Steps 1–5 are repeated *a* times, and the average value of *Frequency*($c_{d}^{i}$, *e_j_*) is calculated as the probability of $c_{d}^{i}$’s being selected to cover *e_j_* (*P_select_*($c_{d}^{i}$, *e_j_*)). Then, each management node m_m_ sends probability *P_select_*($c_{d}^{i}$, *e_j_*) to the corresponding node $c_{d}^{i}$. $c_{d}^{i}$ saves and broadcasts this message (its dynamic assistant event, the corresponding *P_select_*($c_{d}^{i}$, *e_j_*), and the number of its static assistant event |*E_s_*(*s_i_*)|). Each management node *m_m_* receives the message and saves the information.

**Figure 4:**
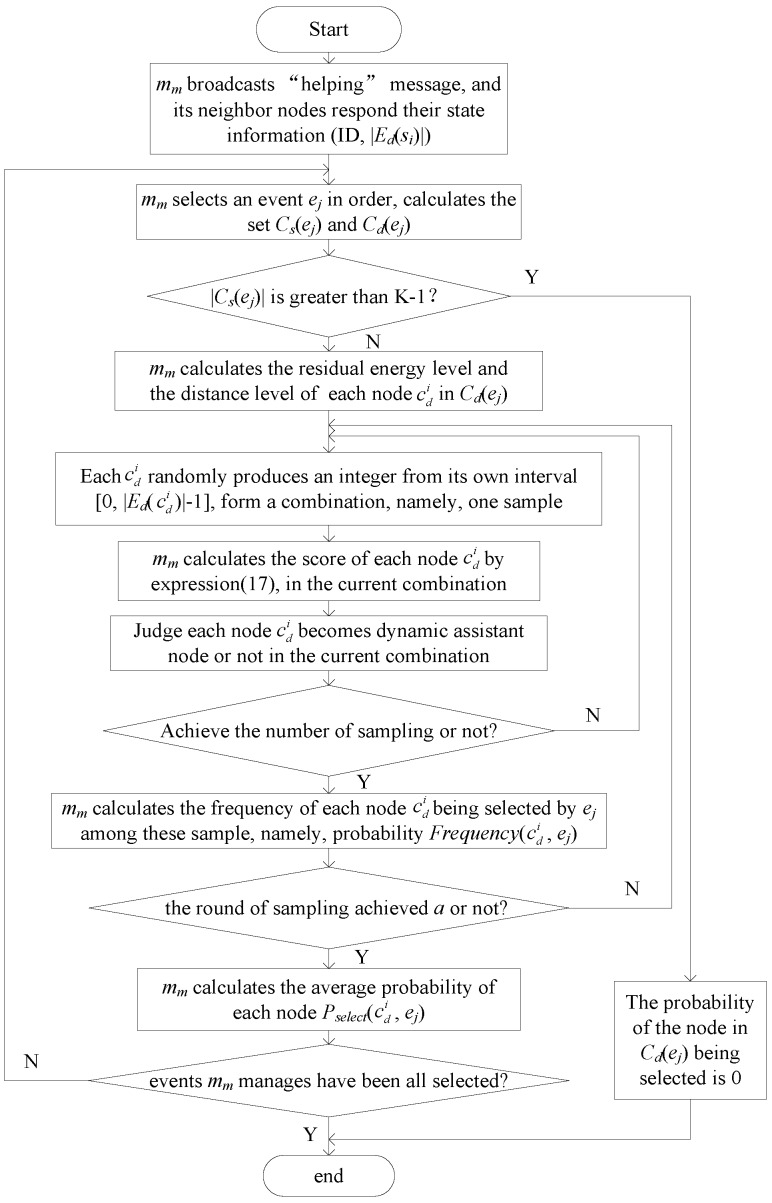
Flowchart of node selection.

The flowchart for calculating the probability of node selected by event is shown in Figure 4, with management *m_m_* as an example.

#### 3.2.3. Multi-Objective Optimization Model

After the process of Section 3.2.3, each management node knows the probability of its neighbor nodes’ being selected by other corresponding events. Each management node (e.g., *m_m_*) then calculates the expectation of the number of other events that its neighbor node (e.g., *s_i_*) may cover ${\bar{E}}_{ad}\left(m_{m},s_{i}\right)$:

(18) $${\bar{E}}_{ad}\left(m_{m},s_{i}\right)=\sum_{e_{j}\in E_{d}\left(s_{i}\right)-E_{M}\left(m_{m}\right)}P_{select}\left(s_{i},e_{j}\right).$$

In Equation (18), *P_select_*(*s_i_*,*e_j_*) is the probability of *s_i_*’s being selected by *e_j_*; in other words, *P_select_*(*s_i_*,*e_j_*) is the probability that *s_i_* assists the corresponding management node in covering *e_j_*. Thus, the sum of the probability of events that *s_i_* can cover, except what *m_m_* manages, is the average number of events that *s_i_* actually covers, namely, the expectation of the event *s_i_* actually covers.

Meanwhile, *m_m_* calculates the corresponding expectation of *s_i_*’s sensing radius ${\bar{R}}_{s}\left(m_{m},s_{i}\right)$. This problem is equivalent to the question of calculating the expectation of *s_i_*’s sensing radius, with all known possible sensing radii of the node *s_i_* and corresponding probabilities.

After *m_m_* learns the number of events that its corresponding neighbor node covers and that the other management nodes manage, it builds optimization models according to the information, the residual energy of the node, and the distance to the corresponding event. For the event *m_m_* manages and whose static assistant node number is greater than K − 1, *m_m_* need not consider it for the adjusting strategy because the events have been K-coverage without any nodes adjusting the sensing radius. Thus, the event is removed out of *E_M_*(*m_m_*) before the optimization models are built.

Assuming that *x_ij_* denotes whether *s_i_* is selected to cover *e_j_* or not, and that *b_ij_* expresses whether *e_j_* is the dynamic coverage event of *s_i_* or not (for *m_m_*, if *d*(*s_i_*,*e_j_*) is known, then *b_ij_* is also known),

$$x_{ij}=\left\{ \begin{array}{l} 0\;s_{i}\;\mathrm{does}\;\mathrm{not}\;\mathrm{cover}\;e_{j}\\ 1\;s_{i}\;\mathrm{covers}\;e_{j} \end{array}\right.,\;b_{ij}=\left\{ \begin{array}{l} 1\;R_{s}^{\min}<d\left(s_{i},e_{j}\right)\leq R_{s}^{\max}\\ 0\;else \end{array}\right..$$

If *x_ij_b_ij_* equals 1, then *s_i_* is selected to adjust its sensing radius to cover *e_j_*, namely, the dynamic assistant node of *e_j_*, which is also the solution of the multi-objective optimization model.

Therefore, the energy consumption, the residual energy variance, and event detection performance are described after the strategy of the node adjusting sensing radius.

##### Energy Consumption

**Figure 5:**
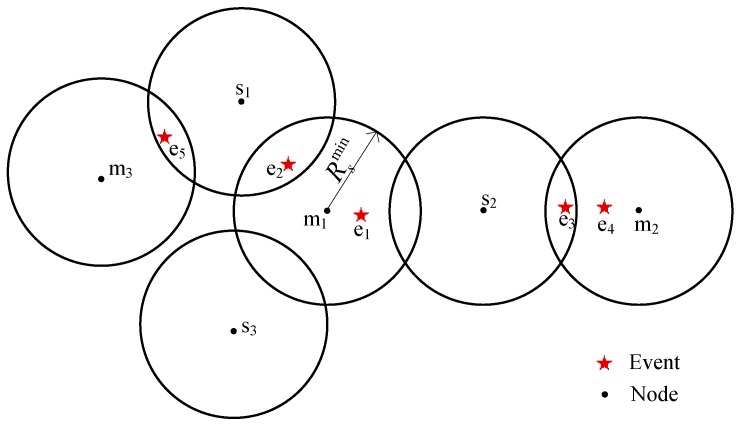
Schematic diagram.

At first, the process whereby each management node (e.g., *m_m_*) calculates the energy consumption of its neighbor nodes (e.g., *s_i_*) with three cases is analyzed as follows:

(1) *s_i_* dynamically covers one event or more, namely, *s_i_* dynamically covers the event *m_m_* manages, or its ${\bar{E}}_{ad}\left(m_{m},s_{i}\right)$ is not equal to 0; then, *m_m_* calculates the energy consumption of *s_i_* produced by the cover of these events that *m_m_* manages. For example, for the node *s*_2_ in Figure 5, its detecting energy consumption is *ECS*(*R*_2_) in the network, but *m*_1_ calculates its energy consumption produced by covering *e*_1_; in other words, the *ECS*(*R*_2_) is divided into two parts by *e*_1_ and *e*_4_. Because *m*_1_ does not know whether *s*_2_ covers *e*_4_ or not, in this study, the number of events that other nodes manage and that *s*_2_ covers is estimated by the expectation of the actual coverage event number ${\bar{E}}_{ad}\left(m_{1},s_{2}\right)$. Therefore, *s*_2_’s energy consumption that *m*_1_ calculates is $ECS\left(R_{2}\right)/\left(1+{\bar{E}}_{ad}\left(m_{1},s_{2}\right)\right)$.
(2) *s_i_* only covers some static events, while *m_m_* calculates the energy consumption of *s_i_* produced by the cover of these events that *m_m_* manages. In Figure 5, the energy consumption of *s*_1_ is *ECS*($R_{s}^{\min}$) and is divided into two parts by *e*_2_ and *e*_5_. Because the static cover event number of *s*_1_ is definite, the *s*_1_’energy consumption *m*_1_ calculates is $ECS\left(R_{s}^{\min}\right)/\left(1+1\right)$.
(3) *s_i_* does not cover any event, and the total energy consumption of *s_i_* is calculated. In Figure 5, *s*_3_’s energy consumption *m*_1_ calculates *ECS*($R_{s}^{\min}$).

(Assuming that *m*_1_ manages events *e*_1_ and *e*_2_, *m*_2_ manages events *e*_3_ and *e*_4_, and *m*_3_ manages *e*_5_, *s*_2_ dynamically covers events *e*_1_ and *e*_4_, *s*_1_ only covers events *e*_5_ and *e*_2_, *s*_3_ does not cover any events, the sensing radius of *s*_1_ and *s*_3_ are both $R_{s}^{\min}$, and the sensing radius of *s*_3_ is *R*_2_).

The sensing energy consumption of its neighbor nodes is the sum of the energy consumption of each neighbor node and formulated as follows:

(19) $$Energy\left(m_{m}\right)=\sum_{s_{i}\in S_{N}\left(m_{m}\right)}r_{energy}\left(s_{i}\right)\cdot ECS\left(R_{i}\right)$$

where *R_i_* is the current sensing radius of *s_i_*, whose value is the maximum among ${\bar{R}}_{s}\left(m_{m},s_{i}\right)$ and is the distance to the event it covers in *E_M_*(*m_m_*). When both of them are zero, *R_i_* is the minimum sensing radius. The expression for *R_i_* as follows:

(20) $$R_{i}=\max\left(R_{s}^{\min},x_{ij}b_{ij}d\left(s_{i},e_{j}\right),{\bar{R}}_{s}\left(s_{i}\right)\right)\;j=1,2,\ldots \left|E_{M}\left(m_{m}\right)\right|.$$

*r_energy_* (*s_i_*) is the proportionality coefficient, and its expression under the three case above is formulated as follows:

(21) renergy(si)={∑j=1|EM(mm)|xijbij(E¯ad(mm,si)+∑j=1|EM(mm)|xijbij)if E¯ad(mm,si)+∑j=1|EM(mm)|xijbij≠0k2|Es(si)|if E¯ad(mm,si)+∑j=1|EM(mm)|xijbij=0, and |Es(si)|≠01if E¯ad(mm,si)+∑j=1|EM(mm)|xijbij=0, and |Es(si)|≠0

where *k*_2_ is the number of events that *m_m_* manages; meanwhile, *s_i_* can also cover statically. In the expression, the first item corresponds to Case (1), where the denominator expresses the number of events *s_i_* dynamically covers in the whole network and the numerator expresses the number of events *s_i_* dynamically covers in the event set *E_M_*(*m_m_*). Similarly, the second item corresponds to Case (2), and the last one corresponds to Case (3).

Thus, the total sensing energy consumption of the network approximates to the sum of the energy consumption that each management node calculates, denoted by Equation (19).

##### Residual Energy Variance

The residual energy variance of neighbor nodes of *m_m_* is *Variance*(*m_m_*) after the adjusting strategy runs one round.

(22) $$Variance\left(m_{m}\right)=\sum_{s_{i}\in S_{N}\left(m_{m}\right)}{\left(RE_{after}\left(s_{i}\right)-{\bar{RE}}_{after}\right)}^{2}$$

where ${\bar{RE}}_{after}$ is the average residual energy of *m_m_*’s neighbor nodes after *m_m_* uses the adjusting strategy, and *RE_after_*(*s_i_*) is the residual energy of *s_i_*, which can describe the residual energy before using the adjusting strategy *RE*(*s_i_*)minus for the energy consumption of sensing and communication in the current round. The formulation as follows:

(23) $$RE_{after}\left(s_{i}\right)=RE\left(s_{i}\right)-ECS\left(R_{i}\right)-bool\cdot ECC\left(R_{c}\right)$$

where *bool* is a Boolean parameter, which determines if *s_i_* should transmit information to *mm*. Its formulation as follows:

$$bool=\left\{ \begin{array}{l} 1\;x_{ij}b_{ij}=1\;or\;d\left(s_{i},e_{j}\right)<R_{s}^{\min}\\ 0\;else \end{array}\right..$$

This equation indicates that, when the node is selected as the dynamic assistant node of *e_j_* or the node statically cover *e_j_*, the node should detect *e_j_* and transmit information about *e_j_* to mm.

##### Event Detecting Performance

The detecting performance of all events in *E_M_*(*m_m_*) (*Performance*(*m_m_*)) is equal to the sum of that individual event:

(24) $$Performance\left(m_{m}\right)=\sum_{e_{j}\in E_{M}\left(m_{m}\right)}P_{result}\left(e_{j}\right).$$

In the process of calculating the detecting performance, the assistant node set of each event *e_j_* (*A*(*e_j_*)) is formulated as follows:

(25) $$A\left(e_{j}\right)=\left\{a|a=x_{ij}b_{ij}s_{i},i=1,2,\ldots \left|S_{N}\left(m_{m}\right)\right|\right\}\cup C_{s}\left(e_{j}\right)-\left\{0\right\}.$$

The element in *A*(*e_j_*) is the ID of the assistant node of *e_j_*, consisting of the dynamic assistant node (the first item in the equation) and the static assistant node (the second item in the equation). Because the first item includes the element 0, which does not belong to the node ID, the set with the element 0 is removed (the last item in the equation).

The multi-objective optimization model that each management node *m_m_* builds is discussed as follows:

Objective 1: The energy consumption of *m_m_* neighbor nodes is minimized.

$$\min\;Energy\left(m_{m}\right).$$

Objective 2: The residual energy variance of *m_m_* neighbor nodes is minimized.

$$\min\;Variance\left(m_{m}\right).$$

Objective 3: The detecting performance of the event m_m_ that manages is maximized.

$$\max\;Performance\left(m_{m}\right).$$

Some constraint conditions also exist, which are as follows:

Constraint condition 1: Each event must cover at least K − 1 assistant nodes.

$$\left|C_{s}\left(e_{j}\right)\right|+\sum_{i=1}^{\left|S_{N}\left(m_{m}\right)\right|}x_{ij}b_{ij}\geq K-1\;j=1,2,\ldots \left|E_{M}\left(m_{m}\right)\right|.$$

Constraint condition 2:

*x_ij_* = 0, 1.

#### 3.2.4. Methodology of the Constrained NSGA-II and Optimization Strategy

NSGA-II [38] is the improved version of the NSGA. It adopts a fast non-dominated sorting procedure, a crowded comparison operator, and a controlled elitism mechanism, and overcomes certain disadvantages in the NSGA, such as high computing complexity, premature convergence, and the requirement of an assigning sharing parameter. The progress of solving the multi-objective optimization model in Section 3.2.3 is shown as follows (if the space of the multi-objective optimization model is less than the number of population *n_p_*, then the solution set is solved by an exhaustive method directly and ignores the following steps):

(1) Encoding the solution

**Figure 6:**
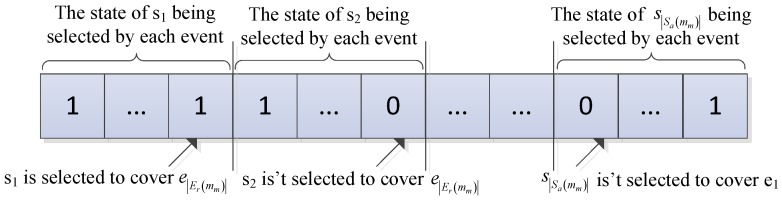
Solution encoding figure.

To select the best node set in the neighbor nodes of each management node *m_m_*, the solution can be expressed as bit string *b_s_* (*b_s_* [1] is the first element in *b_s_*, and *b_s_* [2] is the second element in *b_s_*). Specific coding is shown in Figure 6, and the bit number of *b_s_* is |*S_N_*(*m_m_*)|·|*E_M_*(*m_m_*)|.

Then,

(26) $$x_{ij}=b_{s}\left[\left(i-1\right)\cdot \left|E_{M}\left(m_{m}\right)\right|+j\right].$$

(2) Fast Non-Dominated Sorting Procedure

After the value of each solution in the current generation is obtained, the rank of each solution is distributed by a fast, non-dominated sorting procedure. The rank of some non-feasible solutions may be greater than that of feasible solutions. Thus, the case may degrade the convergence speed of the algorithm. Therefore, during the rank distribution for each solution, this study adds the following two rules [39]:

1. Feasible solutions are greater than non-feasible solutions.
2. In non-feasible solutions, the rank of solution with a lower degree of restriction is greater.

(3) Selection, Evolution, and Recombination [39]

Selection involves choosing excellent individuals from one generation to implement evolution operation. This study uses the championship method to select parent individuals. Evolution includes crossover and mutation, and this study uses two-point crossover. Recombination involves eliminating inferior individuals to form new species groups of the same scale, according to a situation of parent-and-son generations. This study uses the recombination method in Reference [40].

The population in each generation produces a population of the next generation by using Steps (2) and (3). The method ends until the generation value achieves the maximum iteration number.

After NSGA-II obtains the Pareto solution, this study uses the TOPSIS method [41] to obtain the best strategy according to the energy consumption of detection, the residual energy variance, and the event detecting performance.

## 4. Algorithm Analysis

The message and time complexity of the DEEKA are evaluated in this section.

### 4.1. Message Complexity

The total number of sent and received messages determines message complexity. At the beginning of every round of the algorithm, each dynamic assistant node at the last round broadcasts its residual energy, and the nodes that receive the message save and update the state information of its neighbor nodes. The average number of dynamic assistant nodes in each round is assumed as *n_d_* (*n_d_* ≤ *N*), and the average number of neighbor nodes is assumed as *n_a_*. In this stage, the number of sent and received messages are *n_d_* and *n_d_*·*n_a_*, respectively. In the stage of management node formulation, the node broadcasts message *M_c_* after it detects an event. The number of sent messages is *n_c_* (*n_c_* is the number of nodes detecting the event; *n_c_* ≤ *N*), and the number of received messages is *n_c_*·*n_a_*. Each management node broadcasts a “help” message after its formulation. Other nodes receiving this message broadcast their state message. In the two processes, the number of sent messages is *n_m_* + *n_r_* (*n_m_* is the number of management nodes in every round, *n_m_* ≤ *N*; and *n_r_* is the number of nodes receiving the “help” message, *n_r_* ≤ *N*), and the number of received messages is *n_a_*·(*n_m_* + *n_r_*). Then, each management node sends the message about the probability of a node’s being selected to the corresponding node after the probability has been calculated. Then, each node broadcasts its probability of being selected by the event. In the two processes, the number of sent and received messages is *n_m_* + *n_r_* and *n_a_*·(*n_m_* + *n_r_*), respectively. In the last stage, each management node broadcasts to direct the corresponding nodes to adjust their sensing radius when it has calculated the best strategy. In this process, the number of sent messages is *n_m_*, and the number of received message is *n_a_*·*n_m_*.

Therefore, the total number of sent messages for every round is expressed as follows:

(27) $$n_{d}+n_{c}+2\left(n_{m}+n_{r}\right)+n_{m}\leq 5N.$$

The complexity of sent messages is *O*(*N*).

The total number of received messages for every round is expressed as follows:

(28) $$n_{d}\cdot n_{a}+n_{a}\cdot n_{c}+2n_{a}\cdot \left(n_{m}+n_{r}\right)+n_{a}\cdot n_{m}\leq 5N^{2}.$$

The complexity of received messages is *O*(*N*^2^).

### 4.2. Time Complexity

In management node formulation, all nodes that detect the same event compete to formulate the final management node of the event. The worst aspect of the process is that all of the nodes detect all of the events. In this situation, every node for each event calculates the score of all of the nodes about the event. Thus, the time complexity of the process is *Z*·*N*. In the process of calculating the probability of the node’s being selected by events, each management node synchronously calculates the probability of its neighbor nodes’ being selected by each event by a sampling and statistics method. According to Reference [37], when the number of combinations is greater than 10,000,000, the number of samplings does not exceed 400. Thus, this study sets the sample number regardless of the total number of combinations. In addition, for a more accurate and reliable result, this study samples at round *a* and uses the average as the probability of the node’s being selected. The worst aspect of the process is that all the events are managed by a management node, and other nodes are the neighbor nodes of the management node. In this situation, time complexity does not exceed *MN*(400*a*K + 1). In the process of solving the multi-objective optimization model, the time complexity of NSGA-II is $Ο\left(3n_{p}^{2}\right)$, where *n_p_* is the population number. Finally, each management node selects the best strategy from the Pareto solution set by the TOPSIS method. The TOPSIS method in this study does not need to sort for all the results and selects only the best one. Assuming that the number of Pareto solution sets is *n_s_*, the time complexity of the process is *n_s_*.

The time complexity of the whole process of algorithm *T_c_* is shown as follows:

(29) $$T_{c}=ZN+ZN\left(400aK+1\right)+3n_{p}^{2}+n_{s}\leq \left(400aK+3\right)ZN+3n_{p}^{2}.$$

Because *n_p_* is the constant relative to *Z* and *N*, the time complexity can be expressed as *O*(*ZN*).

## 5. Simulation and Performance Analysis

In this study, the analysis of the DEEKA will be divided into two parts. At first, the validity of the DEEKA is verified for the four indicators, namely, event detecting performance, network energy consumption, network reliability, and energy consumption balance, compared to an algorithm of the same class, the OVSKA. Then, the DEEKA is compared to an algorithm of another class (DMNSA [26]) in terms of network energy consumption, event detecting performance, and the event detecting delay to verify the correctness of the analysis.

### 5.1. Simulation Scenario and Parameter Settings

A UWSN’s event K-coverage process is simulated by maxtrix laboratory (MATLAB) based on the background of Zhejiang offshore breeding sites, in which the speed of the water current is relaxed, underwater terrain is relatively flat and has little impact on the capability of communication and sensing of the node, and there is enough deep to deploy the nodes. Therefore, during the simulation, the target water area (length × width × depth) is set to 100 m × 100 m × 100 m.

After the target water area is determined, the network achieves 1-coverage by using deterministic deployment of the truncated octahedron (the method needs the minimum number of nodes), in which the number of nodes is 560. The network then is simulated according to the description of Section 2.1.1. Because the paper focuses on the application effectiveness of the algorithm on event K-coverage, the paper assumes an ideal physical layer, a MAC layer, and error-free communication links for simplicity, namely, nodes can communicate with each other when they are in the communication range and all of the data bags each node sends or receives are right, but may be lost in a certain probability in the communication process, and the probability *p_lost_* is set to 0.2. For the energy consumption during the network running, the paper use of the network energy model mentioned in Section 2.1.2 calculates the energy consumption of each node. Meanwhile, the paper uses the detection performance model to calculate the performance of the network by the algorithm running.

For the value of each indicator, it is produced by an average of 30 rounds of simulation data. The parameters are set in Table 1 as follows:

### 5.2. Simulation Example

#### 5.2.1. Comparison with the OVSKA

**Figure 7:**
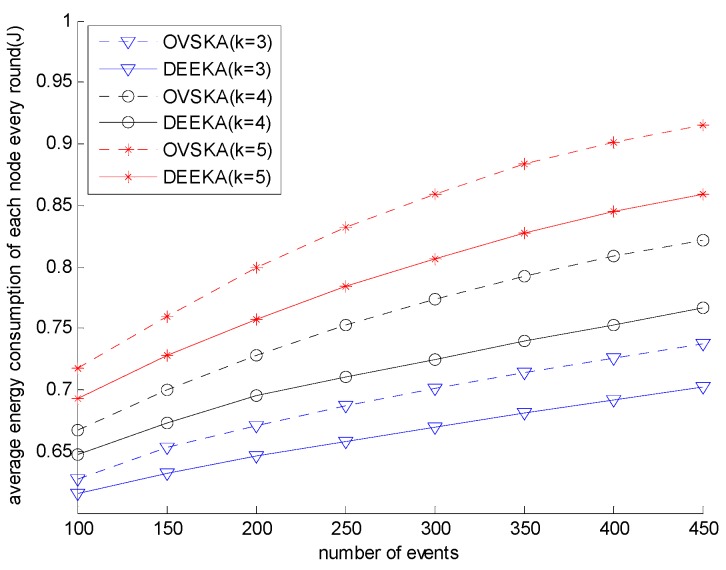
Average energy consumption of each node with event number and K-value.

Figure 7 presents the average energy consumption of each node of both the OVSKA and the DEEKA in every round with the varying number of events under different coverage requirements. In Figure 7, under the same coverage requirements, the energy consumption of both the OVSKA and the DEEKA increases. However, the trend gradually slows down with the increasing number of events. When the number of events is also the same, the energy consumption of the DEEKA is lower than that of the OVSKA. In addition, under different coverage requirements, the energy consumption of the two algorithms also increases. In the DEEKA, each management node selects the assistant nodes considering the expectation number of actual dynamic coverage events of the node. When selecting the assistant node for one event, a situation in which the node is selected by other dynamic coverage events is considered. The process will reduce the number of dynamic assistant nodes and the energy consumption to a certain extent. It will also slow down the increasing speed of the number of dynamic assistant nodes with the increasing number of events or the increasing value of K. On the contrary, the OVSKA does not consider the above situation, which leads to the number of events or the value of K having a relatively great influence on energy consumption.

**Figure 8:**
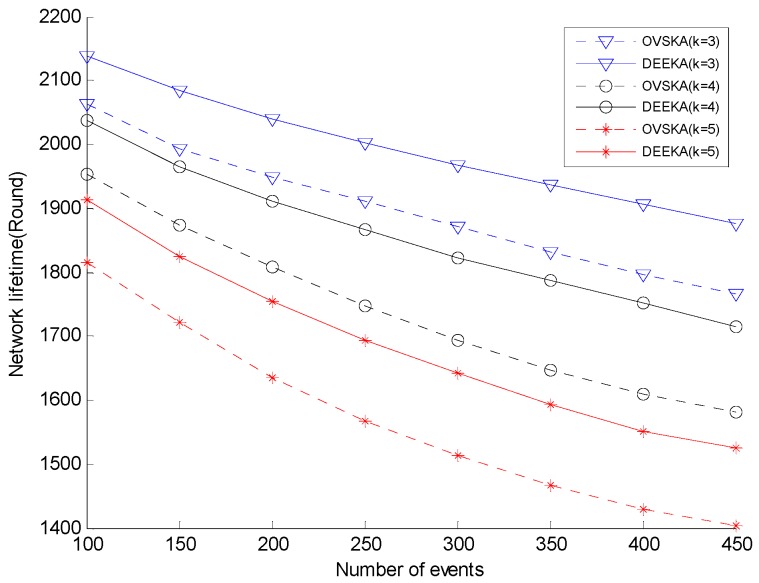
Network lifetime with event number and K-value.

Figure 8 presents the network lifetime of each node of both the OVSKA and the DEEKA with the varying number of events under different coverage requirements. As shown in Figure 8, the network lifetime of both algorithms decreases with the increasing number of events. With the same coverage requirements, the decreasing speed of the DEEKA is less than that of the OVSKA, and when the number of events is the same, the network lifetime of the DEEKA is greater than that of the OVSKA. In addition, the decreasing trend of the network lifetime of the DEEKA is less than that of the OVSKA. In the OVSKA, each management node ignores the residual energy of the node and situations in which a node is selected by several dynamic coverage events when it selects the assistant nodes. The process produces several dynamic assistant nodes in the network and frequently makes some nodes the dynamic assistant nodes, which is not beneficial to prolonging network lifetime. However, the DEEKA considers these situations. In the DEEKA, a node can cover several dynamic coverage events to a great extent, which can slow down the increasing speed of network energy consumption. In addition, both the management node formulations of each event and the selection of the dynamic assistant node in the DEEKA considers the residual energy of the node, which can balance network energy consumption and is beneficial to prolonging network lifetime.

Figure 7 and Figure 8 shows the trend of each indicator with the varying number of events and the varying value of *K*. The trend of each indicator from the network starting to run to the network ending will be shown in this section. The number of events is set 250, and the value of *K* is set 4.

**Figure 9:**
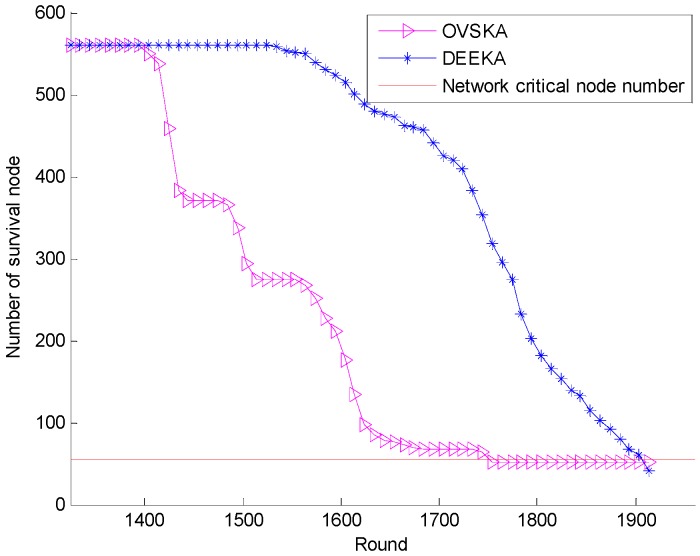
Number of survival nodes with the number of network running rounds.

Figure 9 presents the number of survival nodes with the network running round. As shown in Figure 8, both the running time where none of the nodes died and the network lifetime are greater in the DEEKA. Moreover, the number of survival nodes reduces slowly at first, and the trend then increases gradually when the first dying node appears. In addition, the number of survival nodes of the DEEKA under the same round is greater than that of the OVSKA. In the OVSKA, each management node ignores the residual energy of the node when it selects the assistant nodes, which makes some nodes become the dynamic assistant nodes frequently and then accelerates them to die. However, in the DEEKA, each management node regards the residual energy variance of its neighbor nodes as one of the optimization objectives to select its assistant nodes. Specifically, it considers the residual energy of its neighbor node during the process, which can balance network energy consumption to slow down node death and prolong network lifetime. In addition, in the DEEKA, each management node calculates the energy consumption of its neighbor node, considering the expected actual dynamic coverage event of the node, which reduces the energy consumption of the network. Thus, the process is also beneficial to slowing down node death.

**Figure 10:**
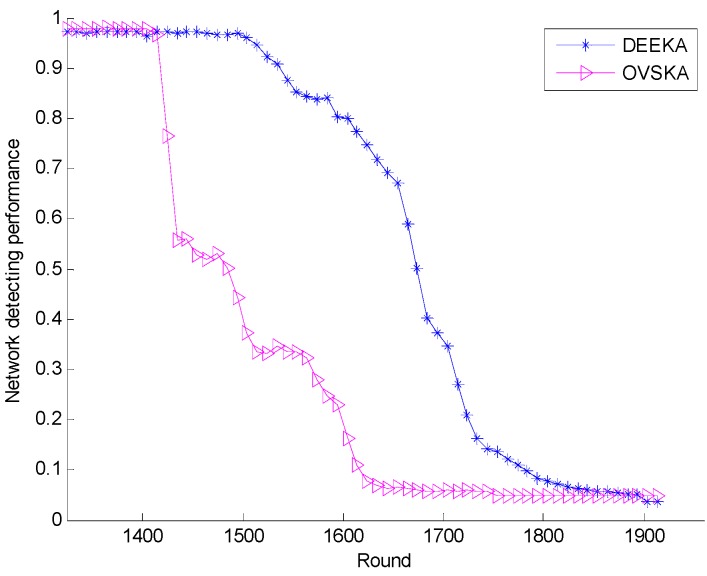
Network detecting performance with the number of network running rounds.

Figure 10 presents the network detecting performance of both the OVSKA and the DEEKA with the network running round. When nodes did not die in the network, the network detecting performance of the OVSKA was better than that of the DEEKA with a slight difference. However, when the dying nodes appear, the network detecting performance of the OVSKA decreases quickly, and that of the DEEKA decreases slowly and maintains a relatively good performance in a certain round. In the DEEKA, each management node selects the assistant node considering the balance of the energy consumption of the node, which can reduce the speed of node death and maintain a relatively high number of survival nodes in certain rounds, as shown in Figure 9.

**Figure 11:**
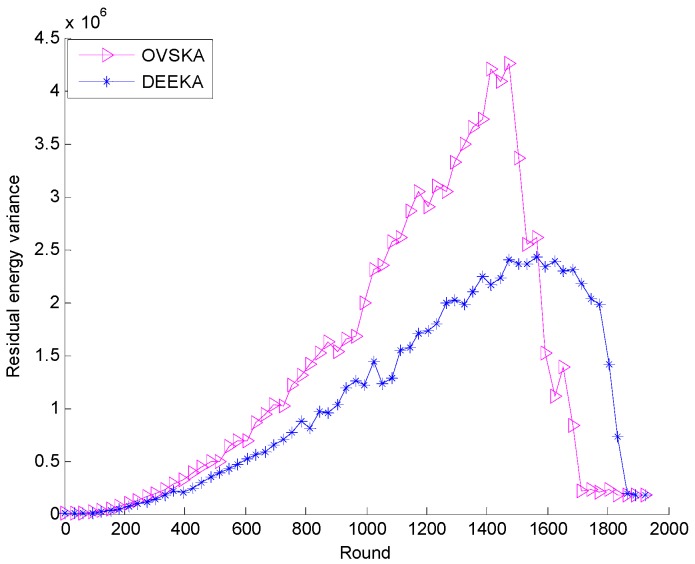
Residual energy variance with the number of network running rounds.

Figure 11 shows the residual energy variance of both the OVSKA and the DEEKA with the network running round. As shown in Figure 10, the variance of both algorithms increases, but the increasing trend of the OVSKA is greater than that of the DEEKA. Additionally, they decrease quickly with the decrease in the number of survival nodes. This phenomenon benefits from each management node with regard to residual energy variance of its neighbor nodes as one of the optimization objectives in the DEEKA, that is, considering the residual energy of its neighbor when it selects the assistant nodes and making network energy consumption more balanced.

**Figure 12:**
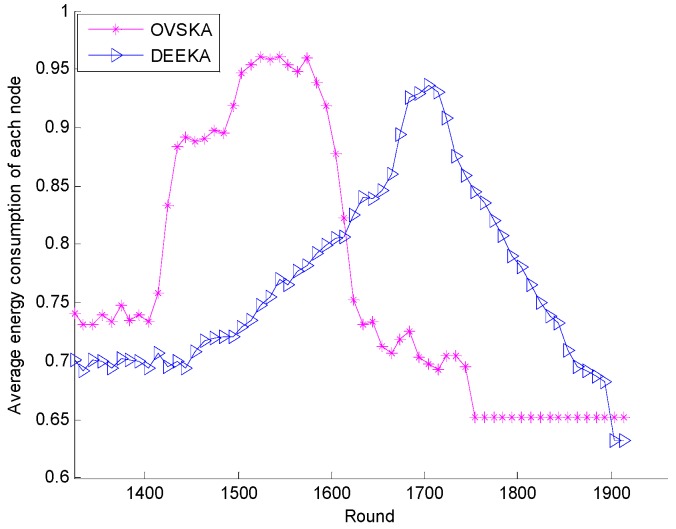
Average energy consumption of each node with the number of network running rounds.

Figure 12 shows the average energy consumption of each node of the OVSKA and the DEEKA with the network running round. In Figure 12, the network energy consumption of the two algorithms in the network without node death moves down and up among certain values, but the energy consumption of the DEEKA is less than that of the OVSKA. Because in the DEEKA, each management node selects the assistant node considering a situation in which the node may be selected by several dynamic coverage events, which can reduce the number of dynamic assistant nodes to a certain extent and reduce network energy consumption. When the death node appears in the network, the average energy consumption of both algorithms increases at first and then decreases, but the increasing trend of the DEEKA is less than that of the OVSKA because the number of survival node decreases as round number increases, which makes the average sensing radius of the node increase in turn. In other words, network energy consumption increases. When the number of survival nodes decreases to a certain value, dynamic assistant nodes do not exist for some events. Thus, energy consumption decreases. The DEEKA considers the balance of network energy consumption, thereby slowing down the death rate of the node and maintaining the number of survival nodes at a high level for a period of time, thereby slowing down the increasing rate of the average sensing radius of the node, i.e., slowing down the increasing speed of the network energy consumption.

#### 5.2.2. Comparison with the DMNSA

The DEEKA is compared to another class of algorithm in this section. For the kind of algorithm used for static K-coverage for the area, the K-coverage for the area, regardless of whether a sleep strategy of the node is used, should be completed. Clearly, the number of nodes that the K-coverage network needs is K times as much as that 1-coverage needs. Thus, this study does not compare the DEEKA to this kind of algorithm because the result is obvious. For the kind of algorithm that assisted mobile nodes, we chose instead the distributed mobile node selection algorithm (DMNSA) proposed by reference [26] for event K-coverage using mobile nodes. The position of the mobile node is randomly distributed in the area, and the number of mobile nodes is equal to the number of nodes in the network for K-coverage. Meanwhile, the energy consumption of moving unit distance is 1.2 J [26].

**Figure 13:**
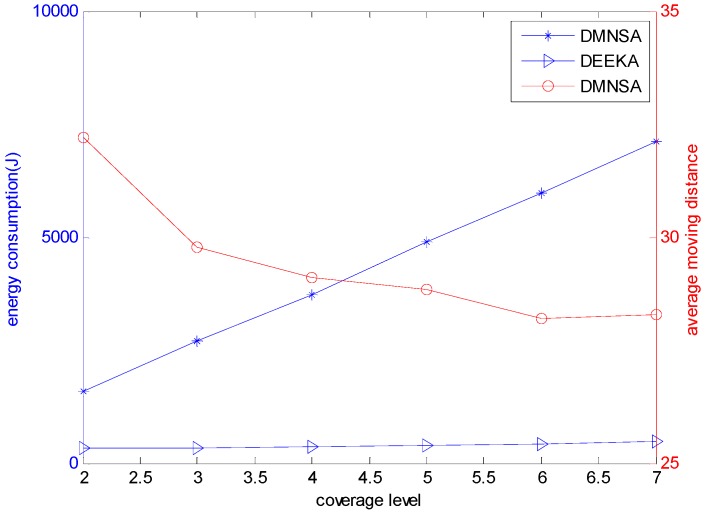
Energy consumption and average moving distance with varying K.

Figure 13 presents the energy consumption of the DEEKA and the DMNSA, and the average moving distance of the DMNSA with the varying value of K. In Figure 13, energy consumption increases with increasing coverage level, but the trend of the DEEKA is less than that of the DMNSA. Meanwhile, the energy consumption of the DEEKA is less than the DMNSA with the same coverage level. The reason is that the DEEKA completes the event K-coverage by adjusting the sensing radius of the node, and the sensing energy consumption is much lower than the energy consumption of the moving node. In addition, the average moving distance of the node decreases as coverage level increases. However, the node still needs some time to move to the destination because of the low moving speed of the node in water [29]. Contrarily, the method of adjusting the sensing radius that the DEEKA uses only needs to adjust the transmit power of the sensor, which is completed instantaneously.

**Figure 14:**
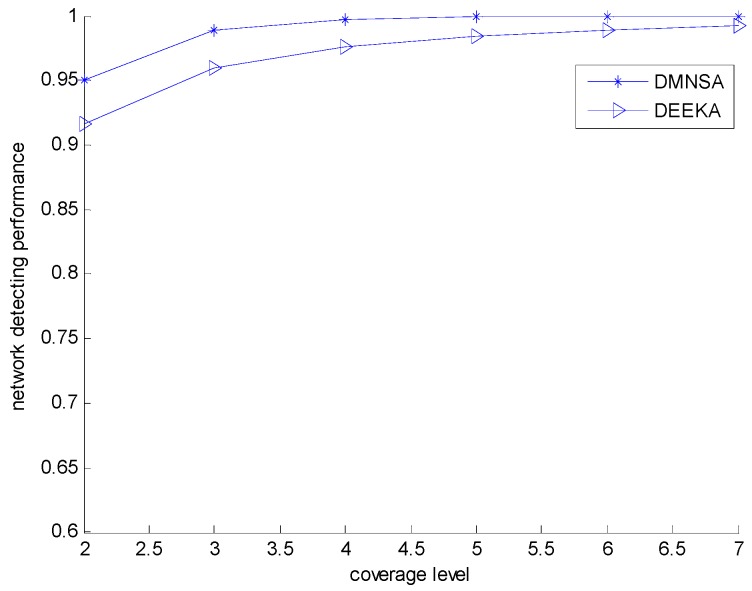
Network detecting performance with varying K.

Figure 14 shows that the network detecting performance of the DEEKA and the DMNSA with the varying coverage level. In the figure, the network detecting performance of the DEEKA is less than that of the DMNSA, but the difference closes with increasing coverage level and the greatest difference between them is also no more than 5% because the DMNSA uses mobile nodes to complete K-coverage and the event is all within the minimum sensing radius of the node. However, the DEEKA adjusts the sensing radius to complete the event K-coverage, and, for each event, there are some nodes among the K nodes that need to increase their sensing radius to cover the event, which degrades the detecting quality of the event. When the coverage level increases, its impact on the network detecting performance, caused by the increasing the sensing radius, weakens gradually. That is to say, the robustness of the network increases.

## 6. Conclusions

This study presents the DEEKA and considers the effect of harsh underwater environments on information collection and transmission. It also considers the residual energy of the node and a situation in which the node is selected by several other events. At the beginning of each round of the algorithm, the nodes that detect the same event compete for the management node of the event with the number of neighbors and the average residual energy, as well as distance to the event. Then, each management node calculates the probability of each dynamic candidate node’s being selected by the corresponding event it manages according to the levels of distance and the residual energy of nodes in the set of dynamic candidate nodes, as well as the number of dynamic coverage events of the node. Afterwards, each management node builds a multi-objective optimization model of the expected energy consumption of its neighbor nodes, the residual energy variance of its neighbor nodes, and the detecting performance of the events it manages as targets. Then, the management node obtains Pareto solutions by using the constrained NSGA-II method. Finally, *m_m_* selects the best strategy using the TOPSIS method, according to target bias of practical application. Simulation results show that, unlike the OVSKA, the DEEKA balances and reduces network energy consumption, thereby prolonging the network’s best service quality and lifetime.

In future work, we plan to find a more precise method to establish the multi-objective programming model combining the characteristics of UWSNs. In addition, we will try to find a special or simple method to solve the model to further optimize our algorithm.

## Figures and Tables

**Table 1:** Simulation parameter table.

Parameter	Value	Parameter	Value
Information transmission failure *p_lost_*	0.2	Minimum sensing radius $R_{s}^{\min}$	10 m
Energy consumption of data reception *P_r_*	5 mW	Maximum sensing radius $R_{s}^{\max}$	28 m
Data transmission speed underwater	1000 bit/s	Communication radius *R_c_*	20 m
Interval of algorithm operation *T*	6 s	Length of data packet *l*	150 bit
Adjusting parameters $\gamma_{1},\gamma_{2}$	0.23, 0.71	Energy diffusion factor *λ*	1.5
*k*_1_, *a*	1, 10	Carrier frequency *f*	24 kHZ
Ratio of sensing to communication power *r_p_*	43/80	Maximum iteration number	300
Values of weight *w*_1_,*w*_2_,*w*_3_	1/3, 1/3, 1/3	Number of population *n_p_*	60
